# Does the Novel Integrated PET/MRI Offer the Same Diagnostic Performance as PET/CT for Oncological Indications?

**DOI:** 10.1371/journal.pone.0090844

**Published:** 2014-03-06

**Authors:** Jiahe Tian, Liping Fu, Dayi Yin, Jinming Zhang, Yingmao Chen, Ningyu An, Baixuan Xu

**Affiliations:** 1 Department of Nuclear Medicine, General Hospital of the Chinese People's Liberation Army, Beijing, China; 2 Department of Radiology, Xiyuan, General Hospital of the Chinese People's Liberation Army, Beijing, China; The George Washington University, United States of America

## Abstract

**Background:**

We compared PET/MRI with PET/CT in terms of lesion detection and quantitative measurement to verify the feasibility of the novel integrated imaging modality for oncological applications.

**Methodology/Principal Findings:**

In total, 285 patients referred to our PET/CT center for oncological indications voluntarily participated in this same-day PET/CT and PET/MRI comparative study. PET/CT images were acquired and reconstructed following routine protocols, and then PET/MRI was performed at a mean time interval of 28±11 min (range 15–45 min). PET/MRI covered the body trunk with a sequence combination of transverse T1WI 3D-volumetric interpolated breath-hold, T2WI turbo spin echo with fat saturation, diffusion-weighted imaging with double *b* values (50 and 800 s/mm^2^), and simultaneous PET acquisition over 45 min/5 bed positions. The maximum standardized uptake value (SUVmax) was assessed by manually drawn regions of interest over fluorodeoxyglucose-positive lesions. Among 285 cases, 57 showed no abnormalities, and 368 lesions (278 malignant, 68 benign and 22 undetermined) were detected in 228 patients. When stand-alone modalities were evaluated, PET revealed 31 and 12 lesions missed by CT and MRI, respectively, and CT and MRI revealed 38 and 61 more lesions, respectively, than PET. Compared to CT, MRI detected 40 more lesions and missed 8. In the integrated mode, PET/CT correctly detected 6 lesions misdiagnosed by PET/MRI, but was false-negative in 30 cases that were detected by PET/MRI. The overall diagnosis did not differ between integrated PET/MRI and PET/CT. SUVmax for lesions were slightly higher from PET/MRI than PET/CT but correlated well (*ρ* = 0.85–0.91).

**Conclusions/Significance:**

The novel integrated PET/MRI performed comparatively to PET/CT in lesion detection and quantitative measurements. PET from either scanner modality offered almost the same information despite differences in hardware. Further study is needed to explore features of integrated PET/MRI not addressed in this study.

## Introduction

To a certain extent, clinical management of oncological patients relies on medical imaging to detect, stage, and monitor the size, site, biological activity and response to treatment of tumors. The increasingly expanding choices of anti-tumor therapy and the trend of personalizing clinical oncology therapy has motivated enhanced medical imaging technology in previous decades. Multimodality imaging, which integrates anatomical/structural and functional/metabolic information derived from computed tomography (CT) and positron emission tomography (PET), was introduced and gradually recognized as valuable since the late 1990s [1]–[3]. Inspired by the success of PET/CT, a new type of integrated imaging device, PET/magnetic resonance imaging (PET/MRI), was commercially available in 2010, and immediately drew intensive interest from the worldwide medical community [4]–[8]. Theoretically, the combination of MRI and PET, which possesses good soft-tissue contrast, good flexibility in acquisition parameters for tissue characterization and little radiation exposure (MRI)as well as increased sensitivity, numerous radiolabeled tracers for various *in vivo* molecular targets (PET), is desired in clinical as well as research applications [4]–[8].

However, despite a number of preliminary reports in favor of the added value by combining data from PET and MRI in neurology, cardiology and oncology [9]–[14], the new power of this integrated system has not been fully validated clinically. Clinical validation of the integrated PET/MRI for routine use is mandatory because previous research pointed to problematic mutual interference when PET was installed in the MRI gantry. For example, the high magnetic field alters the positron range and disables the photon multiplier tube (PMT); the radiofrequency pulse might cause spurious counts [15], [16], and the component of the PET detector might jeopardize the homogeneity of the magnetic field and produce extra heat. As well, the efficacy and accuracy of attenuation correction based on MRI was an issue [17]–[19], and in particular the new hybrid equipment did not use conventional PET and MRI because manufacturers were forced to use avalanche photodiodes or silicon photomultipliers to make the PET detector smaller and the magnetic field compatible. In addition, the MRI scanner and coils had to be redesigned to adapt to the “inserted” PET detector.

After such a PET/MRI scanner (Biograph mMR, Siemens) was installed in our institute, we initiated a one-to-one comparison of the new hybrid device with PET/CT.

## Methods

### Objectives

Our aim was to validate the clinical feasibility of the integrated PET/MRI for general oncologic application in terms of lesion detection and quantitative measurement by comparing it to PET/CT in a daily-routine clinical setting.

### Patient Population

Patients were selected sequentially from those referred to our PET/CT center for tumor-related indications from May 2012 to February 2013. A total of 303 patients volunteered to undergo same-day PET/CT and PET/MRI: 18 were excluded because of incomplete data or technological reasons; finally 285 patients (171 males) with complete clinical and imaging data were eligible for further analysis. The current study focused on oncology purposes Patients were excluded if they were unable to undergo 2 imaging sessions because of illness or other restrictions (e.g. incompatible metal implant, possible pregnancy, under age 15 years, etc.) or if image quality was unacceptable, mainly caused by strong artifacts on MRI images. The demographic and clinical information for patients is in Table 1.

**Table 1:** Demographic and clinical data of patients.

Total patients	285
Male	171
Age (yr), mean±SD	53.8±12.7
Female	114
Age (yr), mean±SD	51.4±12.2
Lesions	368
Malignant	278
Benign (definitely confirmed)	68 (27)
Undetermined	22
No lesion	57
Referential Indications	
Diagnosis confirm/differentiation	119
Staging and treatment planning	41
Post-treatment monitoring	88
Tumor screening	37

Data are number unless indicated.

### Ethics

The study was implemented at the Chinese PLA General Hospital. All procedures for the study were approved by the Medical Ethics Committee of the hospital, and all patients signed an informed consent before undergoing PET/MRI and PET/CT imaging arranged sequentially at the same visit to our center.

### PET/CT

PET/CT followed our routine protocols. Briefly, the patient fasted for 6 h and rested for at least 20 min in a quiet waiting room before intravenous administration of ^18^F-fluorodeoxyglucose (^18^F-FDG; produced in our institute under good manufacturing practice conditions) at 2.22 to 4.44 MBq (0.08–0.12 mCi)/kg. Patients were asked to continue their comfortable resting position for another 55 to 60 min. Whole-body imaging covered from the chin to upper thigh with 10- to 20-min/5- to 7-bed data collection after low-dose CT scanning (120 kV, 100–120 mA/s, 5-mm slice thickness, 5-mm increment, pitch 1) adjusted by the patient's body weight and height and the scanner (Advance VCT, GE, and Biograph 64, Siemens). As with the routine protocol, no contrast enhancement was used for PET/CT. The images were reconstructed with CT attenuation correction (AC) by use of OSEM software provided by the venders. The 2 PET/CT scanners from different vendors were calibrated as per our daily quality assurance procedures.

### PET/MRI

PET/MRI data were acquired by use of an integrated PET/MRI scanner (Biograph mMR, Siemens) that had a YSO crystal-APD PET detector assembly fixed inside a 3.0T MRI gantry between the body coil and gradient magnet coil. The PET part had a 25.8-cm axial field of view in the z-direction, and the MRI part was equipped with a PET-compatible total image matrix coil. The MRI data and PET data were acquired simultaneously by use of the acquisition protocol recommended by the vender and tailored by a series of phantom tests before the clinical study. Combined MRI sequences covered the body truck and included transaxial 3-D volumetric interpolated breath-hold T1-weighted sequence (T1 3D-VIBE), transaxial T2-weighted sequence turbo spin echo with fat saturation (T2 TSE-FS), transaxial diffusion-weighted image (DWI) sequences with double *b* values (50 and 800 s/mm^2^) acquired after a coronal fast-view T1-weighted localizer sequence and a transaxial 2-point Dixon sequence to generate an MRI-based AC map. Both MRI and PET mages were acquired simultaneously at 5 min per bed position (BP), and the total acquisition took 50∼60 min over 4∼5 BP for each body trunk scan. The data for the head with different sequence complexes were not analyzed in the current study. Breath-hold and diaphragm navigation techniques were used during acquisition over the upper abdomen. For logistic reasons, 271 patients underwent PET/MRI at a mean time interval of 28±11 min (range 15–45 min) after PET/CT scan; only 14 cases underwent PET/MRI first followed by PET/CT. No further radiopharmaceutical therapy was needed for the second imaging session. No contrast enhancement was used in MRI scanning.

### Comparison of PET/CT and PET/MRI

All PET/CT and PET/MRI images were retrospectively reviewed with consensus by 2 physicians with experience in PET or PET/CT (12 and 5 yr, respectively) and MRI (2 and 6 yr, respectively). Any positive lesion in each subject, presenting as abnormal FDG uptake, focus of different density, or abnormal signal of unexplainable nature, for example, was defined and counted. The findings of single modality (i.e., PET from both hybrid scanners, CT, and MR, on T1- or T2-weighted images) were first evaluated and compared, then integrated images from PET/CT and PET/MRI were reviewed and compared. During the image reading, the readers were blinded to the related clinical information. Images with severe artifacts were rejected from further analysis.

Lesions were quantitatively analyzed in the same way for PET/CT and PET/MRI. The maximum standardized uptake value (SUVmax) was obtained in regions of interest (ROIs) drawn over the lesion (or the most representative one with multiple lesions), provided that the lesion was positive for ^18^F-FDG.

The endpoints of this comparative study included lesion detection rate and contribution of each imaging modality to diagnostic gains as well as quantitative measurement. The standard of truth, for positive or negative findings, benign or malignant lesions, was justified by histopathological evidence, therapeutic response after the imaging, or other clinical confirmation (disease manifestation, laboratory findings and other clinical findings) during 9- to 22-month follow-up (median 15 months).

### Statistical Analysis

The detection rate of each imaging modality was compared by chi-square test. Kolmogorov-Smirnov test was used to detect normal distribution of SUVmax derived from the seven ROIs by PET/CT and PET/MRI. Wilcoxon matched-pairs signed-rank test was used to test the mean values of PET_AC_CT_ and PET_AC_MRI_ in non-normally distributed samples. Spearman correlation analysis was used to compare SUVmax for the 2 integrated modalities. SSPS 11.5 (SPSS Inc., Chicago, IL) was used for statistical analysis, and *p*<0.05 was considered statistically significant.

## Results

### Scanning and Findings

All patients successfully completed paired imaging without any side effects. No severe discomfort was reported in PET/CT study. Most patients underwent PET/MRI without remarkable events. About 10% of patients complained of a little dizziness and were tired after PET/MRI. No claustrophobia was encountered; however, because of the much longer scanning time with PET/MRI (up to 70 min in some poorly cooperative cases), 3 relatively older or sick patients could not endure the whole-body study and had to drop out in the middle of the scanning, and 15 others had severe artifacts on PET/MRI images, which were then excluded from the final analysis. To the end of follow-up, lesions were positive for 228 patients and negative for 57. For the 228 positive cases, 368 lesions were identified in various parts of the body. In all, 278 of 368 lesions were found malignant and 68 benign (27 confirmed by surgery); 22 were not determined (Table 2). About one quarter of MRI images had some minor artifacts, such as smears at the upper chest and eyes, ghosts in aortas or gallbladder, and lost signals due to metal implants [19], [20], but these were easily recognized and had no effect on analysis of lesions.

**Table 2:** Comparison of the detection rate of each modality.

	CT(+)	CT(−)	*Chi-square value*	MR(+)	MR(−)	*Chi-square value*
PET (+)	266	35	0.22*	287	14	29.45^#^
PET (−)	39	85		61	63	
CT (+)				297	8	31.34^#^
CT (−)				51	69	

*P>0.05, ^#^ *P*<0.01.

### Lesion Detection Rate

Despite different detector configurations and acquisition parameters, PET images from either system (PET/CT: Biograph 64, Advance VCT; and PET/MRI: Biograph mMR) had exactly the same detection rate of lesions, although the lesion-to-background contrast varied slightly because of the time lag between PET/CT and PET/MRI. PET detected 35 and 14 lesions missed by CT and MRI, respectively, whereas CT and MRI revealed 39 and 61 more lesions than PET alone, respectively. Compared to CT, MRI detected 51 more lesions and missed only 8 lesions. The detection rates of PET and CT did not differ (*p*>0.05), but detection rates for both differed from that with MRI (*p*<0.01). In analysis of fused images for each integrated modality, integrated PET/CT detected 6 more lesions than PET/MRI, and the latter corrected 30 false-negative PET/CT lesions. Both integrated systems revealed no false-positive findings for our 57 true-negative patients (Table 3). In light of the standard of truth, PET/MRI and PET/CT showed similar detection for 332 of 368 lesions (90.2%) and reached identical diagnostic performance for 249 of 285 patients (87.4%).

**Table 3:** Comparison of integrated imaging on a per-lesion basis.

	PET/MRI (+)	PET/MRI (−)	*P value*
PET/CT (+)	332	6	1.00*
PET/CT (−)	30	0	

^*^Considering all lesions, differences in lesion detection was not significant (Fisher's exact test, 2-sided) between PET/CT and PET/MRI (6 false-negative PET/MRI results, including 4 tiny lesions in lungs, 2 bony destructive lesions; 30 false PET/CT results, including 5 small liver lesions, 6 hepatobiliary lesions, 4 pancreatic lesions, 4 renal lesions, 4 post-peritoneal foci, 3 head-and-neck infiltrations, 2 prostate lesions, 1 stomach and 1 colon lesion).

### Performance of Imaging Modalities

**Figure 1:**
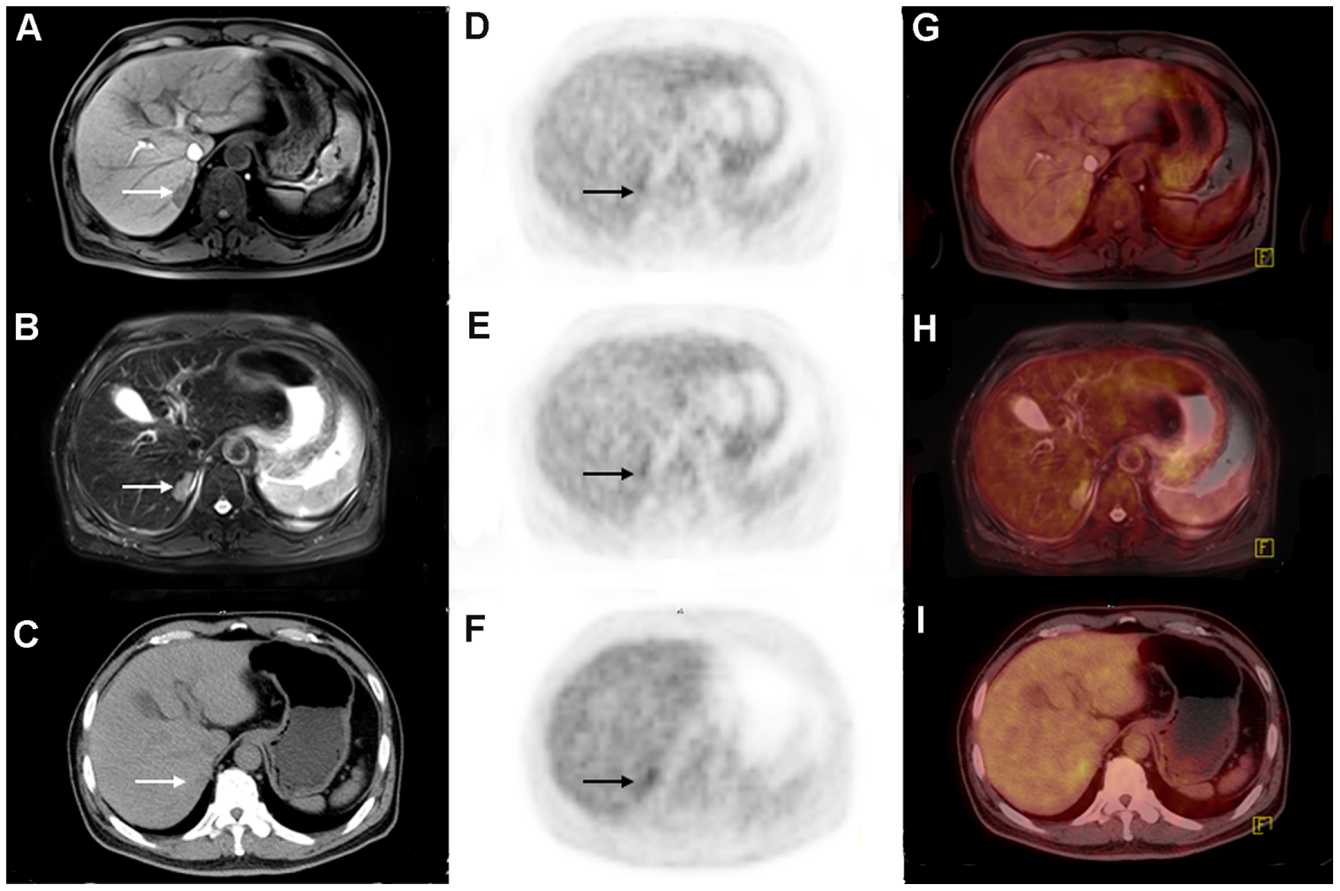
A 62-year-old female with history of breast cancer resection with a solitary liver nodule in follow-up study. T1WI (B), T2WI-FS (D) and CT (F) showed the well-moderate differentiated hepatocellular carcinoma (*arrows*), whereas PET (A, C, and E) did not show abnormal uptake of fluorodeoxyglucose (FDG).

**Figure 2:**
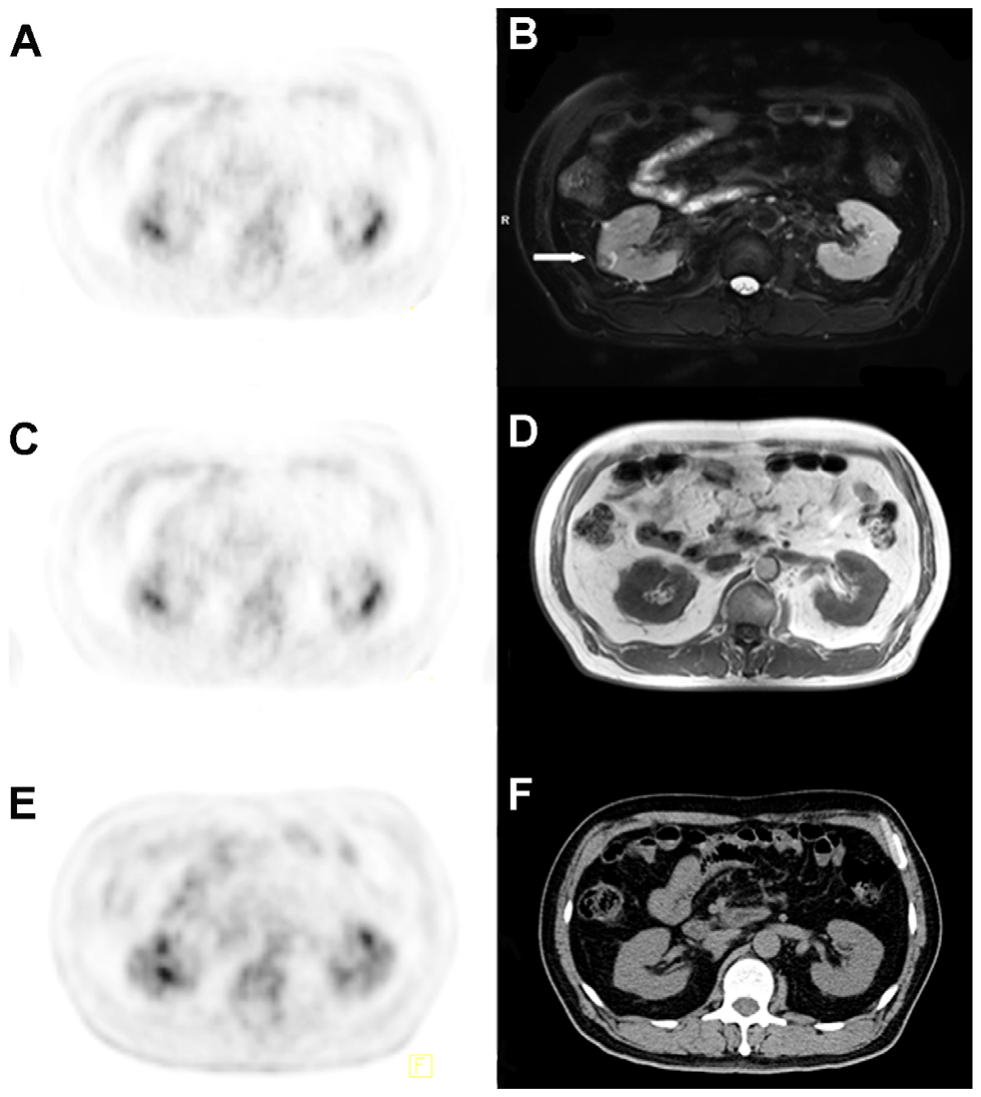
In a 52-year-old male, a right renal nodule was incidentally found by PET/MRI, and the renal lesion was later proven to be a renal cell carcinoma, Fuhrman II grade. T2WI-FS showed the lesion (B, *arrow*) much better than T1WI (D) and CT (F); PET (A, C, and E) was false negative.

**Figure 3:**
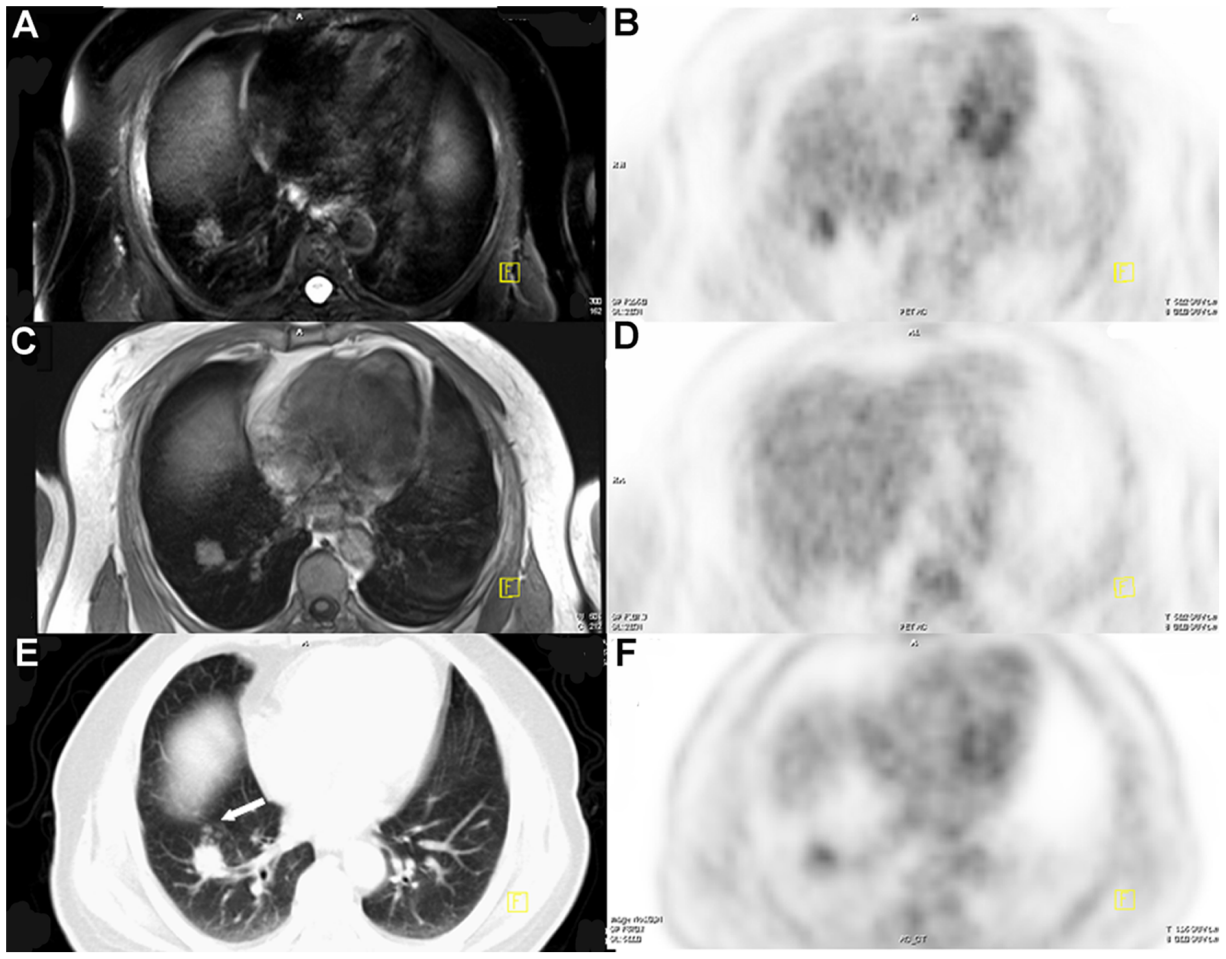
A 67-year-old male underwent imaging for highly suspected lung cancer. T2WI-FS (A), T1WI (C) and CT (E) showed the main lesion at the right lung, whereas small satellite nodules (*arrow*) were detected by CT alone. Mild FDG uptake was observed on PET images (B, D, and F). The lesion was later confirmed to be a tuberculous granuloma.

**Figure 4:**
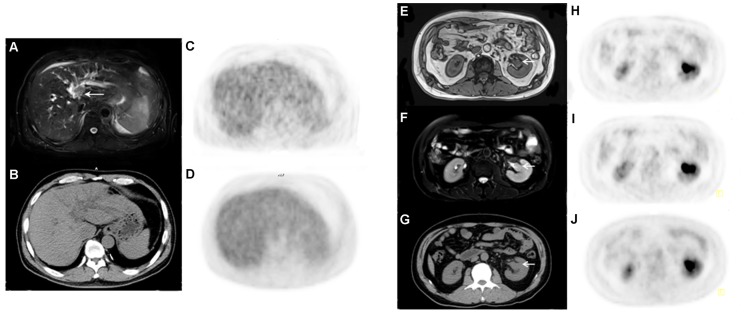
Representative cases of PET/MRI. In a 52-year-old symptom-free male, ultrasonography incidentally revealed a dilated bile duct and CT a dilated common bile duct. T2WI-FS outlined a mass inside the dilated bile duct (*arrow head*), which was later removed by surgery and the lesion was proven to be an epithelial cancer of bile duct origin (A). An right renal pelvic mass was better delineated against hot urine on T2WI-FS in a 60-yr-old woman; an invasive ureteral epithelium carcinoma was later proved by surgery (*cross,* B).

Regarding the differences in lesion detection for the imaging modalities, PET missed 29 benign lesions, 26 malignancies and 12 undetermined lesions. Most of the PET false-negative lesions were small or in regions of high-uptake background (e.g., liver and kidney; Fig. 1) or were certain histological types (e.g., clear cell carcinoma, Fig. 2). CT better illustrated 39 non-FDG-negative lesions that were missed by PET and 8 lesions in the lung and skeletal system that were missed by MRI (Fig. 3) but gave false-negative results for 3 benign, 25 malignant and 25 undetermined lesions. In contrast to CT, MRI was superior in the liver–biliary–pancreas region, kidney, and digestive tract, which helped to correct 61 and 51 PET- or CT-missed lesions, respectively (Table 2, Fig. 4A, 4B). PET/MRI gave false-negative results for 4 tiny pulmonary lesions and 2 bony destructive lesions, and PET/CT gave false results for 5 small lesions in liver, 6 hepatobiliary tract, 4 pancreas, 4 kidney, 4 post-peritoneum, 3 bone marrow, 2 prostate, 1 stomach and 1 colon (Table 3). The lesion detection rate did not differ between PET and CT, but the MRI detection rate of was higher than that with PET and CT (*p*<0.01) The lesion detection rate was same, but if integrated PET/CT and PET/MRI were used, the latter revealed more lesions and more clearly illustrated the lesions against their surrounding tissues in about one third of malignant cases. The above-mentioned diagnostic difference mainly reflected the performance of the CT and MRI component, because the PET findings were the same with either integrated modality. The performance of PET/CT and PET/MRI in various body regions showed that PET/MRI was more powerful than PET/CT in most body parts except the lungs and bones (Table 4).

**Table 4:** Detection rate of PET/CT and PET/MRI in various body regions.

Location of lesions	Feature of lesions (*n* = 346) *		Time difference (mean ± SD, min)	Detection rate of PET/CT	Detection rate of PET/MRI
Head and neck	B	9	38±9	6/9	9/9
	M	23	39±13	23/23	23/23
Lung	B	9	34±6	9/9	6/9
	M	37	35±12	37/37	36/37
Mediastinum	B	11	35±6	11/11	11/11
	M	16	37±12	16/16	16/16
Liver/spleen/biliary tree	B	7	32±3	5/7	7/7
	M	52	33±11	43/52	52/52
Pancreas	B	7	29±9	4/7	7/7
	M	27	34±13	26/27	27/27
Gastrointestinal system	B	1	33	1/1	1/1
	M	21	32±7	19/21	21/21
Kidney/adrenal gland	B	4	29±9	2/4	4/4
	M	15	30±11	13/15	15/15
Pelvic (genital system, prostate, etc)	B	6	28±7	5/6	6/6
	M	15	26±12	14/15	15/15
Bones	B	2	32±4	2/2	2/2
	M	7	33±13	7/7	5/7
Other (retroperitoneal space, soft-tissue, etc.)	B	12	29±14	11/12	12/12
	M	65	34±12	62/65	65/65

*The 22 lesions of undetermined nature were not included in the table.

B: benign lesions.

M: malignant lesions.

### Quantitative Measures

**Figure 5:**
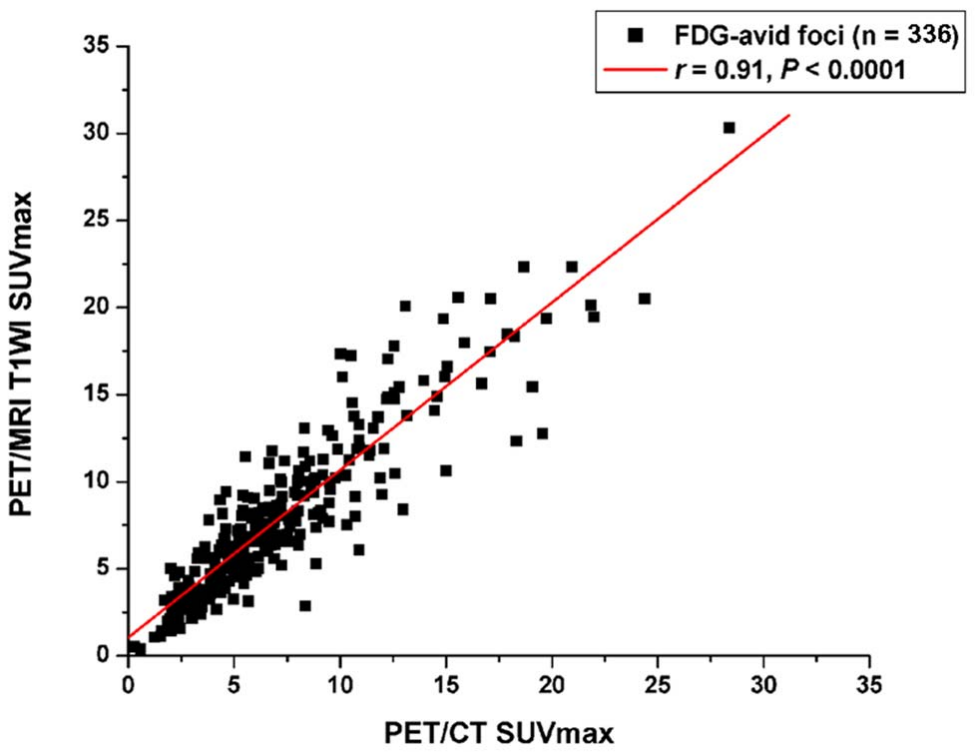
Correlation of FDG-positive foci SUVmax between PET/CT and PET/MRI.

**Figure 6:**
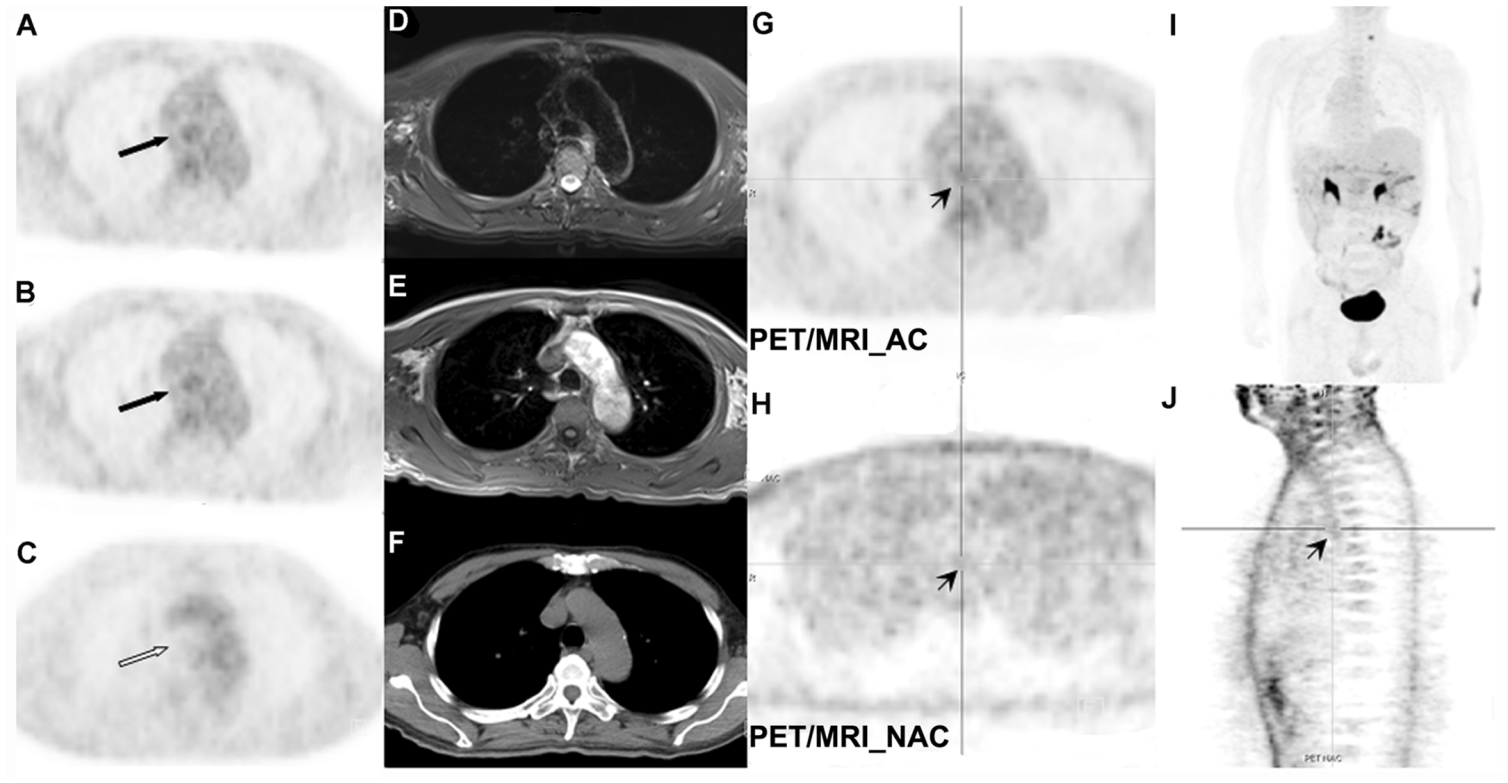
“Hot trachea” artifact on PET/MRI images. The “hot trachea” artifact present on MRI-based FDG-PET images (A and B, *arrow*) compared with CT-based PET image (C). Structural images of T2WI-FS (D), T1WI (E), and CT (F) images showing the corresponding levels of trachea lumen. Although increased radioactivity (*arrow head*) was found in the transaxial MRI-based PET image (G), the maximum intensity projection (I) seemed clear. A similar artifact (*arrow head*) was noted, although less intense, in the non-AC images for the same subject (H and J).

The SUVmax for lesions from PET/CT and PET/MRI, whether benign or malignant, were highly correlated (*ρ* = 0.91; Fig. 5). Both PET/CT and PET/MRI gave significantly higher SUVmax with malignant than benign lesions (7.83±4.79 and 8.49±5.06 vs. 5.94±3.57 and 7.13±3.98, respectively, *p* = 0.000–0.016). The absolute SUVmax were slightly higher with PET/MRI than PET/CT, especially for malignant lesions, which was most likely related to the time lag between the 2 imaging sessions. An unexpected finding with PET/MRI was remarkably higher FDG activity in trachea lumen than with PET/CT (1.84±0.45 vs. 0.99±0.35, *p*<0.001). The “hot trachea” on PET images with PET/MRI (Fig. 6) presented various intensity in nearly two thirds of cases. Although a similar phenomena was present in non-AC PET images (Fig. 6H, 6J), the artifact had no clear explanation, except for improper attenuation correction over a gas-containing cavity with PET/MRI.

## Discussion

### Feasibility of Integrated PET/MRI

The deepening knowledge about human cancers and advances in tumoral therapeutics, driven by the progress in genomics, epigenetics, and new targeted anti-tumor agents, have demanded even better imaging facilities with potential of revealing *in vivo* biological and pathophysiological characteristics of tumors at the molecular level. Inspired by the success of integrated PET/CT, integrated modalities combining morphostructural and functional/metabolic imaging techniques are quickly being developed. As well described in several review articles, the combination of PET and MRI has long been considered promising, because PET has superb sensitivity and potential for targeting a large variety of molecules inside the body, and MRI is favorable in multiparametric imaging flexibility, has excellent soft-tissue contrast and *in vivo* chemical composition detection, and less radiation. An editorial by Mansi, Ciarmiello and Cuccurullo anticipated that PET/MRI could bring us the “third eye” for the yet-to-be-discovered molecular information in addition to the “binocular vision” of morphostructural and functional findings [20]. However, because the 2 modalities are not compatible with each other, PET, MRI and coils needed to be greatly modified in both hardware and software for an integrated model, which was comprehensively reviewed in a dedicated issue of the *European Journal of Nuclear Medicine and Molecular Imaging* in 2009. The US Food and Drug Administration approved the first integrated PET/MRI scanner in 2010, and about 40 such scanners were installed worldwide by the end of 2012. Most of the early users positively reported the usefulness of the new tool for different disorders [21]–[27]. Recently, Wiesmülle et al. reported their comparative study of PET/MRI and PET/CT and concluded fairly good agreement in diagnosis with 3 different radiopharmaceuticals for 43 patients [28]. Nevertheless, most studies were limited in number of cases, which somewhat weakened their power in terms of evidence-based medicine. Therefore, a paired comparison between PET/MRI and PET/CT in a larger cohort of patients with non-specifically restricted oncological indications under a general clinical environment would complement these pioneering efforts to fully validate the feasibility of the novel integrated system. This kind of study could better clarify whether the integrated PET/MRI could be an alternative or competitor to PET/CT for oncological indications, for confidence in use of the knowledge from decades-long experience with PET/CT and for value in paving the road for further application and development of integrated PET/MRI.

### Lesion Detection Comparable Between PET/MRI and PET/CT

In our 285 cases, the largest single-study cohort so far, the detection rate of PET/MRI and PET/CT was similar for a wide spectrum of tumors or non-tumor lesions located in various parts of the body. In per-capital or per-lesion–based comparative analysis, results from both modalities were in agreement for in 332 of 368 lesions (90.2%) and for 249 of 285 patients (87.4%). Those results strongly support the feasibility of integrated PET/MRI as a diagnostic imaging tool, equal to or in certain cases better than PET/CT for general oncological indications.

Two points should be emphasized in reviewing the results. First, the stand-alone modalities (i.e., PET, CT, and MRI) of the integrated instruments complemented each other in lesion detection (Table 3). In a small but significant proportion of the studied cases, one modality might lead to the correct diagnosis that was missed by the other 2 modalities alone. Second, PET with both PET/MRI and PET/CT, although with about a 30-min time lag between them, had similar results. The difference in detection rate with the 2 hybrid modalities mainly reflected the diagnostic power and weakness of their CT and MRI components. CT was better in revealing small nodules in lungs, bones and lesions with calcification, whereas MRI complemented CT for lesions located in morphostructural complex body parts, such as the hepatobiliary–pancreas region, kidney and pelvis. Despite the equal detection rate, PET/MRI provided additional information on the relationship between lesions and adjacent tissue and clearer delineation of the intra-lesion characteristics. This finding is not unexpected because the enhanced value of MRI with respect to CT depends on different “biochemical” components of a tissue rather than purely tissue density or number of cells [20]. The contrast of false-negative results between PET/MRI and PET/CT (6 *vs.* 30) is in keeping with most reports dealing with integrated PET/MRI, PET/CT and stand-alone CT and MRI [9]–[14], [21]–[28]. The better illustration of a lesion and its relation to nearby tissues could have positive impact on clinical decision making, especially surgical planning, but merits further study.

### Quantitative Measurement

At the beginning of the trial, we were concerned about the reliability of SUVmax derived from PET/MRI in view of the unconventional, still-debated AC methods used in PET/MRI [17]–[19], [29]–[31]. From our study, the SUVmax derived from PET/MRI over a large variety of tumors and benign lesions in different parts of the body were in good agreement with those from PET/CT. The absolute SUVmax for lesions slightly differed. This finding is in agreement with Wiesmüller et al. [28], who showed lower SUVmax and SUVmean from PET/MRI than PET/CT, despite good correlations. The differences in SUVmax values in the Wiesmüller et al. study were greater than in ours, which might be caused by the longer time lag between procedures in the previous study than ours (50–166 vs 15–45 min). Although a truncated artifact on µ-map, as described by many investigators, might induce distorted images at the peripheral field of view, the 2-point Dixon sequence-based AC algorithm currently used for PET/MRI may be reliable and free of negative effects on quantitative analysis of most parts of the body, except gas-containing cavities such as the trachea and lungs. These findings were in agreement with other authors [32]–[34], and once again showed the feasibility of use of integrated PET/MRI in structural as well as quantitative imaging, which could be of clinical value in tumor evaluation and therapy monitoring.

### Image Quality and Scanning Protocols

To a certain extent, diagnostic feasibility of integrated imaging depends on the quality of morphological images. The MRI image quality primarily depends on data acquisition [35]. As compared with the stand-alone version, MRI acquisition in integrated PET/MRI agreed with PET acquisition. Our total PET/MRI acquisition time was set to 45 min/5 BPs, but in real practice, extra time was needed in positioning the subject, hooking the coils, shimming the magnetic field after bed movement, and breath-holding cooperation of the subject, which might prolong the scanning time to more than 1 h, thus causing uneasiness or even unbearable discomfort in a few extreme cases. Many combinations of MRI sequence were tried, as introduced by Martinez-Möller et al. [36], to reduce the scanning time, but unfortunately any fast sequence we tried had the unbearable cost of deteriorated image quality. After consulting with MRI experts from local hospitals, a consensus was reached that the current sequence complex was probably the most suitable for us to maintain a reasonable MRI image quality for the general purpose, whole-body tumor imaging.

Artifacts were more frequently encountered with integrated PET/MRI. They had negative effects on MRI images, as described by Hofmann, Keller and others [17], [19], [21], and affected corresponding PET images. One unexpected observation was higher activity inside the trachea lumen, which occurred in two thirds of PET/MRI cases. The quite unusual finding varied in intensity among cases. The cause of such artifacts was not clear and could be related to mis-segmentation of MRI-based AC because of its air content and specific location, as described by Berker [31] and Nuyts [33]. The artifact did not disturb image interpretation but raises some caution that such artifacts might occur elsewhere. This observation calls for further investigation and awareness when interpreting PET/MRI images.

Having reviewed the results of the paired comparison of PET/MRI and PET/CT, the concerns raised by Mansi et al. [20] and others could be positively answered. Even with differences in PET infrastructure and algorithms in image reconstruction and attenuation correction, the diagnostic performance of integrated PET/MRI, including detection of abnormality and quantitative measure, is equal to or even better than that with PET/CT. The artifacts were easily recognized and did not affect interpretation of the results in most cases. Integrated PET/MRI and PET/CT are thus similarly feasible in clinical application, and what we learned from PET/CT could be translated with certainty to interpretation of PET/MRI results.

### Some Other Concerns

Our study differed in study design, conduction and endpoints from most previously reported studies [35]–[40]. We did not focus on specific tumors or on technical issues of integrated PET/MRI but simply tried to validate the diagnostic comparability with reference to the value-proven PET/CT in a rather ordinary clinical scenario. Such a general, one-to-one comparison covering concurrent clinical oncological indications could complement the already reported evidence in showing the feasibility of integrated PET/MRI. However, the longer acquisition time and sophisticated acquisition parameters delayed the though-put to 4 to 5 PET/MRI examinations per day, which is in contrast to that of PET/CT (50–60 cases per day) in our institute. The diagnostic gains of PET/MRI with better delineation of lesions in some patients cannot balance the higher cost of the installation and maintenance of the equipment. Through integration of the 2 most advanced molecular imaging modalities, some exclusive information critical to tumor diagnosis and clinical management might provide solid ground for the new hybrid imaging device. However, we did not investigate the efficacy, management and cost-effectiveness of PET/MRI [41], [42], which needs serious consideration and comprehensive verification.

### Limitations

Without restrictive selection and recruitment of the patients, the studied cohort could not fully demonstrate the clinical powers of PET/MRI (e.g., the protocol used was far from ideal: lack of optimal sequences for certain tumors and body regions, without functional MRI sequences and contrast enhancement) as far as MRI was concerned and for PET (no tumor-oriented tracers other than FDG, and their integration with MRI findings). The number of studied patients was still limited and the effect of non-randomized scanning order had to be carefully considered. Finally, the clinical impact of PET/MRI and PET/CT, in view of the differences in image features and lesion delineation, was not comprehensively assessed and compared. Therefore, the current study was too premature to draw definite conclusions. Detailed, better-designed and more disease-specific study with more patients could reveal the “third eye” of this promising technology.

## Conclusions

This one-to-one comparison of PET/CT and integrated PET/MRI revealed a similar diagnostic performance in lesion detection and quantitative assessment covering a wide spectrum of oncological indications in a routine clinical environment. The differences in the new system in terms of hardware, acquisition, attenuation correction, and image reconstruction algorithm did not affect its diagnostic performance. The new integrated PET/MRI was found as feasible in oncological application as PET/CT. Many unanswered issues such as the impact of clearer tissue/lesion delineation on clinical decision making, the optimization of protocols and parameters, the unexplored features of MRI imaging, and the balance between academic superiority and cost-effectiveness of integrated PET/MRI warrant further exploration.
